# Impact of the free healthcare initiative on wealth-related inequity in the utilization of maternal & child health services in Sierra Leone

**DOI:** 10.1186/s12913-019-4181-3

**Published:** 2019-06-03

**Authors:** Mohamed Boie Jalloh, Abdulai Jawo Bah, Peter Bai James, Steven Sevalie, Katrina Hann, Amir Shmueli

**Affiliations:** 1 Department of Health Management and Economics, School of Public Health, The Hebrew University of Jerusalem, Jerusalem, Israel; 2 34 Military Hospital Wilberforce, Freetown, Sierra Leone; 3 College of Medicine and Allied Health Sciences, University of Sierra Leone, Connaught Hospital, Freetown, Sierra Leone; 4 Sustainable Health Systems, Freetown, Sierra Leone; 5 Australian Research Centre in Complementary and Integrative Medicine, Faculty of Health, University of Technology Sydney, Level 8, Building 10, 235-253 Jones Street, Ultimo, Sydney, NSW 2007 Australia

**Keywords:** Antenatal care, Postnatal care, Inequity, Institutional delivery, Concentration index, Maternal health, Sierra Leone

## Abstract

**Background:**

As a result of financial barriers to the utilization of Maternal and Child Health (MCH) services, the Government of Sierra Leone launched the Free Health Care Initiative (FHCI) in 2010. This study aimed to examine the impact of the FHCI on wealth related inequity in the utilization of three MCH services.

**Methods:**

We analysed data from 2008 to 2013 Sierra Leone Demographic Health Surveys (SLDHS) using 2008 SLDHS as a baseline. Seven thousand three hundred seventy-four and 16,658 women of reproductive age were interviewed in the 2008 and 2013 SLDHS respectively. We employed a binomial logistic regression to evaluate wealth related inequity in the utilization of institutional delivery. Concentration curves and indices were used to measure the inequity in the utilization of antenatal care (ANC) visits and postnatal care (PNC) reviews. Test of significance was performed for the difference in odds and concentration indexes obtained for the 2008 and 2013 SLDHS.

**Results:**

There was an overall improvement in the utilization of MCH services following the FHCI with a 30% increase in institutional delivery rate, 24% increment in more than four focused ANC visits and 33% increment in complete PNC reviews. Wealth related inequity in institutional delivery has increased but to the advantage of the rich, highly educated, and urban residents. Results of the inequity statistics demonstrate that PNC reviews were more equally distributed in 2008 than ANC visits, and, in 2013, the poorest respondents ranked by wealth index utilized more PNC reviews than their richest counterparts. For ANC visits, the change in concentration index was from 0.008331[95% CI (0.008188, 0.008474)] in 2008 to − 0.002263 [95% CI (− 0.002322, − 0.002204)] in 2013. The change in concentration index for PNC reviews was from − 0.001732 [95% CI (− 0.001746, − 0.001718)] in 2008 to − 0.001771 [95% CI (− 0.001779, − 0.001763)] in 2013. All changes were significant (*p* value < 0.001).

**Conclusion:**

The FHCI appears to be improving access to and utilization of MCH services, narrowing the inequity in ANC visits and PNC reviews, but is insufficient in addressing wealth- related inequity that exists for institutional deliveries. If Sierra Leone is to realize a significant reduction in maternal and child mortality rates, it needs to strengthen the effective implementation of FHCI considering incorporating a sector wide approach (SWAp) or a “Health in all Policy” framework to reach the less educated, rural residents and ensuring culturally sensitive quality services.

**Electronic supplementary material:**

The online version of this article (10.1186/s12913-019-4181-3) contains supplementary material, which is available to authorized users.

## Background

Despite the gains in improving the health status of vulnerable segments of the society over the century, inequity in health and healthcare continue to persist globally [1] and obeying the inverse care law – the availability of good quality healthcare seems to be inversely related to the need for it [2]. Such gap in health status between the rich and the poor is prevalent in many developing countries. In recognising the need to bridge the equity gap, most governments and international organisations have included key provisions in their primary healthcare delivery policy initiative to address such disparities [1, 3–5]. Notwithstanding such commitments, the health status among the poor in sub- Saharan Africa is suboptimal [6].

Monitoring trends in equity in health and access to essential health interventions is important in order to tailor scarce public resources to those who are most in need, particularly poor and underserved communities. While low income countries in sub-Saharan Africa face many challenges in collecting and analysing relevant information for observing trends in equity, such challenges which should not be an excuse for inaction [7].

The 2008 Sierra Leone Demographic and Health Survey (SLDHS) identified cost as the main barrier to utilization of maternal and child health (MCH) services and a key contributing factor for the high maternal and infant mortality rates [8]. In order to address the high maternal and infant mortality rates, the government launched the free healthcare initiative (FHCI) for pregnant women, lactating mothers and children under the age of five in April 2010, which eliminates medical fees and provides drugs and treatments at no cost in every public health facility in the country [9, 10]. However, the FHCI remains challenged by increasing demand, low staffing, and stock-outs of essential laboratory equipment (86–97%), other equipment (13–47%) and drugs (12%), resulting in patients being required to pay out of pocket for services falling under the FHCI [11–13]. Although an increasing number of women and children are reportedly utilizing healthcare services, the FHCI may not have eradicated differential distribution of services among the different wealth quintiles [11, 14].

Despite the FHCI, Sierra Leone was unable to meet its target of the millennium development goals 4 and5 (MDG4 and MDG5) –reducing maternal mortality ratio to 450 per 100,000 births and child mortality to 95 per 1000 live births. The FHCI has since entered into the sustainable development goals (SDG) era with significant gaps in the health sector remaining to achieve SDG 3 – health and wellbeing for all [15, 16]. In Sierra Leone, the current neonatal and under-five mortality rates are at 39 and 156 deaths per 1000 live births respectively and the maternal mortality ratio is 1165 death per 10,000 live births [17]. These infant and maternal mortality indices are far short of the 70 deaths per 100,000 live births target set out in the 2030 sustainable development goal agenda [18]. Even though the FHCI has made MCH services free, indirect costs, among other factors, may still contribute to the disparity in the utilization of MCH services. However, little is known on the impact of the FHCI in narrowing the wealth-related inequity in the utilization of MCH services. For instance, studies have reported that despite the FHCI, women in rural communities, many of which are poor, still experience difficulty in accessing health services [10, 11].

Inequity studies are urgently needed to understand the FHCI’s ability to close the gap between wealth quintiles, which will provide evidence to guide policies aiming to reduce inequalities in access to such services in order to achieve universal health coverage in Sierra Leone. Therefore, we aimed to evaluate the change in the utilization of MCH services among wealth quintiles before (2008) and after FHCI (2013) implementation in Sierra Leone. Further analysis is aimed at demonstrating the impact of secondary factors that affect utilization of MCH services such as education level, residence, ethnicity, age, occupation, religion and number of children of respondents.

## Methods

### Settings

Sierra Leone, which is a low-income country, is approximately 71,740 km^2^ land area divided into four administrative regions namely Northern, Southern, Eastern provinces and the Western area where the capital Freetown is located. The country has a long historical and geopolitical context of poverty, high illiteracy rate. Sierra Leone is also a country that is recovering from disasters including the prolonged 11-year civil war that ended in 2002, followed by the 2012 Cholera outbreak [19] and of recent the 2014–2016 Ebola Virus disease epidemic [20].

Sierra Leone is a low-income country with a reported Gross National Income (GNI) per capita (current dollar, purchasing power parity (PPP) of $1690 while the gross domestic product (GDP) growth rate was 6% in 2013 and the Human Development Index rank for Sierra Leone is 177 out of 187 countries [21]. It has an estimated 2015 population of 7075,64 [22] and the nature of its geography poses significant challenges for the delivery of health services to the population in some of these districts. Sierra Leone currently faces a triple burden of diseases (communicable diseases, 70%; NCDs, 22% and injuries, 7%) [23] common to a growing number of LMICs with life expectancy for both male and female at 50 years [24].

### Data source and sample size

This study was based on the secondary analysis of data obtained from two nationally representative household surveys that interviewed a total of 7374 and 16,658 women of reproductive age (15–49 years) in 2008 [8] and 2013 [25]. Response rates among eligible individuals in the target samples were 94% [8] and 97.2% [25] in 2008 and 2013 respectively.

### Sampling method of SLDHS

All the two Sierra Leone Demographic and Health Surveys (SLDHS) used a multi-stage cluster sampling technique [8, 25]. Initially, the Enumeration Areas (EA) — a cluster that conventionally encompasses 85 adjacent households each were selected as primary sampling units from the sampling frame developed based on the 2004 Census [26]. In each of the selected EAs, a complete listing of households was carried out from which secondary sampling units were drawn using systematic random sampling technique. In the two surveys, 353 EAs were sampled of which 145 were urban and 208 were rural, with each EA having 85 households from which 22 were selected in the second stage of the two-stage sampling [8, 25]. For this study, all data collected from women who gave birth in the preceding 5 years of the survey were included. In cases, where women had more than one birth in the reference period, the most recent one was considered. An algorithm of the number of women interviewed in each of the SLDHS and the women included in the final analysis of antenatal care (ANC) & postnatal care (PNC) (Additional file 1).

### Data analysis

Data analysis were done using Excel Microsoft Corporation and SPSS Package version 22 (SPSS, Inc. Chicago). This study first explored the background characteristics of study participants and then the analysis of MCH utilization by wealth quintile and other individual characteristics. An unadjusted and adjusted binary logistic regression was run for institutional delivery and a concentration curve with subsequent concentration indices generated for ANC visits and PNC reviews for 2008 and 2013 SLDHS.

For MCH utilization variables, we defined the number of antenatal visits (ANC) and post-natal reviews made (PNC) as discrete variables; we considered the number of visits to be complete if it reached the recommended number of visits as per the WHO guidelines [27, 28] (four or more for ANC and four or more for PNC). For ease of analysis, ANC was transformed into three subcategories (none, up to four and more than four visits) and PNC into two subcategories (incomplete and complete). Complete includes all four reviews: post-delivery, prior to discharge, a week after discharge, and 6 weeks post-delivery. If any of these visits were missed, then that constitutes an incomplete PNC. We defined Institutional delivery as the use of a healthcare institution for delivery for the pregnancy under review, regardless of the package of care provided as a binary categorical variable (Yes vs No). We defined wealth quintiles as poorest (1st quintile); poorer (2nd quintile); middle (3rd quintile); richer (4th quintile); and richest (5th quintile). Additional covariates were defined as categorical i.e. education level, occupation, residence (rural/urban), ethnicity, religion, and mother’s age as well as discreet (number of children) variables. All the independent variables were categorical variables except for number of children, which was a quantitative variable.

The undermentioned operational definitions of the dependent and independent variables (see Additional files 2 and 3) were the same as defined in the DHS dataset except for PNC (a composite variable) ethnicity and religion, which were redefined to suit the study design.

The concentration curves were built using two key variables: the independent wealth index variable on the one hand and maternal & child health services utilization outcome variables on the other hand (ANC& PNC). The concentration indices estimated the magnitude of wealth related inequality in the selected MCH services utilization.

During analysis, the cases were grouped according to wealth quintiles into: Poorest: 1st quintile; Poorer: 2nd quintile; Middle: 3rd quintile; Richer: 4th quintile; Richest: 5th quintile.The sum of each outcome variable noted for the five wealth quintiles and then expressed as a percentage of the total outcome variable of interest. Each curve, therefore, represents the cumulative percent of the outcome variable of interest against the cumulative percent of the wealth quintile of the sample analyzed. If ANC visits or PNC reviews utilization were equally distributed across the different wealth quintiles, a 45-degree line representing perfect equality would be generated. This line known as the line of equality (LOE) runs from the bottom left corner of the graph (0,0) to the upper right corner of the graph (100, 100) [29]. If these services were however utilized more by the rich than the poor, the curve falls below the LOE and the further it is away from the LOE the more the wealth-related inequality in the distribution of the MCH services utilization. Since the aim was to compare the wealth related inequality in ANC visits or PNC reviews utilization across a period using the 2008 and 2013 SLDHS, the concentration curves for each outcome variable were plotted on the same graph. Thus, if the curve of one of the time periods (2008 vs 2013) lies above the other (closer to the LOE), then the former is said to dominate the latter, but the extent is unknown. In order to get an exact measure of the degree of inequality, a concentration index is built from each curve and it is defined as double the area between the curve and the LOE [29]. The concentration indexes obtained were then used to rank these two-time periods by the degree of inequality. If the two curves cross each other, a case of non-dominance may be demonstrated.

In this study, the concentration index was calculated first as twice the area between the curve and the line of equality. However, since the area under-the-curve approach to calculating the confidence interval (CI) does not give the standard error of the curve and hence the CI, the CIs were therefore computed using the convenient regression method. The CI was computed in the convenient regression method as twice the weighted variance of fractional living standard variable squared (δ^2)^ and the health variable (h_i_ = ANC or PNC) divided by the mean of the health variable (μ) based on the left hand of eq. 1 below:

1 $$ {2\updelta}^2\left({\mathrm{h}}_{\mathrm{i}}/\upmu \right)=\upalpha +{\upbeta \mathrm{r}}_{\mathrm{i}+\upvarepsilon \mathrm{i}} $$

The computation of the fractional rank of wealth index (r_i)_ was based on equation below for the weighted data.

2 $$ {\mathrm{r}}_{\mathrm{i}=\Sigma\ \left(\mathrm{Wj}+\mathrm{Wi}/2\right)} $$

r_i_ was then sorted in ascending order and its variance calculated. β produced during the convenient regression of the CI variable against the fractional rank variable represents the unadjusted estimate of the concentration index generated on the right hand of eq. 1.

The standardized or adjusted estimate of the concentration index was computed using SPSS statistical software using the generated model to predict the health variable (ANC or PNC) based on eq. 3 below:

3 $$ {\mathrm{Y}}_{\mathrm{i}}={\mathrm{b}}_{\mathrm{o}}+{\mathrm{b}}_1{\mathrm{x}}_1+{\mathrm{b}}_2{\mathrm{x}}_2+{\mathrm{b}}_3{\mathrm{x}}_3 $$

Yi represents the predicted health variable. During the adjustment or standardization of the wealth variable for the other covariates, the adjusted values were predicted using eq. 3 while keeping all covariates at their mean values.

In order to calculate the standard error of the standardized estimate of the concentration index, the sampling variability was taken into account, and thus the convenient regressions were run without transforming the dependent health variable but instead using the transformed living standard variable (i.e. RWealthi).The standard error of the adjusted concentration index was estimated as the coefficient of the transformed living standard variable (RWealthi).The variance of the fractional rank, which was also used in the transformation, depended only on the sample size and so has no sampling variability. It can be treated as a constant. This way the sampling variability was considered because the estimate and its standard error were written as a function of regression coefficients based on eqs. 4, 5, and 6 below.

4 $$ {\mathrm{h}}_{\mathrm{i}}={\upalpha}_1+{\upbeta}_1{\mathrm{r}}_{\mathrm{i}}+{\mathrm{u}}_{\mathrm{i}} $$

5 $$ {}_{\dot{\mathrm{B}}=}\ {\left({{2\updelta}_{\mathrm{r}}}^2/\upmu \right)}_{\dot{\mathrm{B}}} $$

6 $$ {}_{\dot{\mathrm{B}}=}\ {\left[{{2\updelta}_{\mathrm{r}}}^2/\left({\upalpha}_{1+\dot{\mathrm{B}}/2}\right)\right]}_{\dot{\mathrm{B}}} $$

An unadjusted and adjusted binary logistic regression were run to identify how wealth in relation to the other independent variables serves as a predictor of utilization of healthcare institutions for delivery. The generated model predicts whether a pregnant woman will deliver in a health facility or at home based on her wealth index and other independent variables. Logistic regression models were used to obtain unadjusted and adjusted odds ratios with 95% confidence interval for the associations between the different independent variables and institutional delivery. The significant standardized contribution of each covariate was assessed using the adjusted Wald test to obtain the *p*-value. All *p*-values < 0.05 were considered statistically significant.

### Ethical considerations

The DHS program-ICF International, (Rockville, USA), granted access to the data after a submission of a written request through their online platform. The Sierra Leone Ethics and Scientific Review Committee granted a waiver since this is a secondary analysis of de-identified data.

## Results

### Sociodemographic characteristics

The results in Table 1 show that of the women included in the analysis, 75 and 66% had no formal education in 2008 and 2013 respectively; about 70% were rural residents in both 2008 and 2013; about 80% were Muslims in both 2008 and 2013; and 55 and 50% of children had one to four siblings in 2008 and 2013 respectively.

**Table 1:** Weighted Number of Study Participants by Sociodemographic Characteristics 2008 & 2013

	Weighted number of study partipcipants by sociodemographic charactetristics 2008						Weighted number of study partipcipants by sociodemographic charactetristics 2013					
Background characteristics	ANC 2008 Freq (*n* = 3346)	Percent (%)	PLOD 2008 Freq (*n* = 4053)	Percent (%)	PNC 2008 Freq (*n* = 3504)	Percent (%)	ANC 2013 Freq (*n* = 7478)	Percent (%)	PLOD 2013 Freq (*n* = 8625)	Percent	PNC 2013 Freq (*n* = 7971)	Percent (%)
Wealth Index												
Poorest	721	22	885	22	665	19	1667	22	1901	22	1663	21
Poorer	707	21	849	21	623	18	1524	20	1809	21	1550	19
Middle	748	22	893	22	690	19	1556	21	1797	20	1527	19
Richer	629	19	793	19	798	23	1491	20	1694	20	1849	23
Richest	541	16	683	16	728	21	1240	17	1447	17	1382	17
Education level												
None	2510	75	3051	74	2441	70	4920	66	5768	67	5233	66
Primary	411	12	515	13	497	14	1079	14	1203	14	1085	14
Secondary	386	12	482	12	515	14	1374	18	1559	18	1540	19
Higher	39	01	55	01	51	02	105	01	117	01	114	01
Occupation												
Yes	2577	77	3122	77	2585	74	5596	75	6476	75	5791	73
No	769	23	950	23	919	26	1882	25	2148	25	2181	27
Residence												
Urban	1036	32	1183	29	1267	36	2075	28	2387	28	2586	32
Rural	3238	68	2920	71	2236	64	5404	72	6260	72	5385	68
Ethnicity												
Temne,Loko, & Limba	1589	48	1898	46	1369	39	3283	44	3724	43	3211	40
Mende, Sherbro & Kono	1192	36	1512	37	1598	46	3156	42	3714	43	3460	43
Others Sierra Leonean & Foreign	565	17	687	17	536	15	1039	14	1183	14	1300	17
Religion												
Christianity	636	19	794	19	883	25	1388	19	1590	18	1581	20
Islam	2667	80	3247	79	2593	74	6067	81	7005	81	6372	80
Others	43	01	50	01	27	01	24	0.3	25	0.3	18	0.2
Mother’s age												
19–15	270	08	330	08	290	08	751	10	859	10	824	10
24–20	664	20	804	20	722	08	1527	20	1773	21	1683	21
29–25	953	29	1213	30	1018	21	1853	25	2142	25	1945	24
34–30	579	17	704	17	614	29	1421	19	1644	19	1493	19
39–35	559	17	673	16	553	16	1152	15	1354	16	1250	16
40–44	209	06	251	06	208	06	485	07	554	06	491	06
45–49	111	03	127	03	98	03	290	04	322	04	285	04
Siblings												
None	707	21	871	21	737	21	1811	24	2112	24	1948	24
1–4	1863	56	2269	56	1935	55	3631	50	4166	49	3835	49
> 4	776	23	96	23	832	24	2036	26	2369	27	2188	27

### MCH services utilization rates

Table 2 highlights MCH services (ANC, Institutional Delivery and & PNC reviews) utilization rates in 2008 and 2013. Although more than 50% of women attended the four ANC visits recommended by WHO focus antenatal care guideline in 2008, this number increased to 75% in 2013. Institutional delivery among women respondents increased from 27% in 2008 to 57% in 2013. There was also a reduction in the number of incomplete postnatal visits from 92% in 2008 to 59% in 2013.

**Table 2:** Weighted Profile Distribution of MCH Services Utilization in 2008 & 2013

MCH Services	Distribution	2008	2013
ANC	None	8.2%	2.2%
	Up to four visits	41%	22.6%
	More than four visits	51%	75.2%
Institutional delivery	Yes	27%	57.2%
	No	73%	42.8%
Postnatal reviews	Complete	8.3%	41.4%
	Incomplete	91.7%	58.6%

### Inequality analysis of ANC visits

**Fig. 1:**
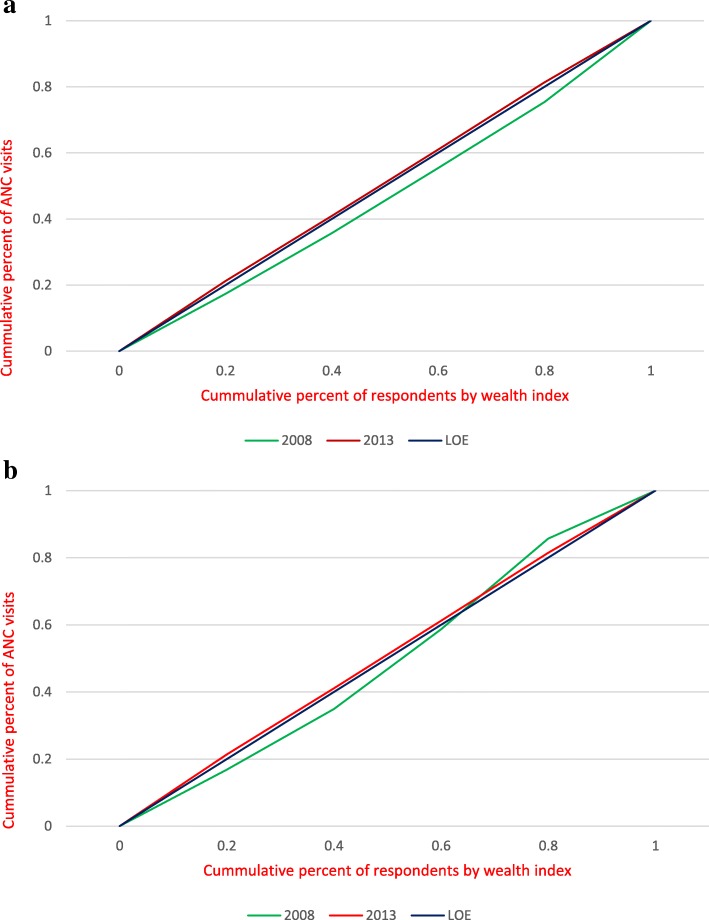
**a** Weighted Unadjusted Concentration Curves for ANC visits in 2008 and 2013 SLDHS. **b** Weighted adjusted concentration curves for ANC visits in 2008 and 2013 SLDHS

The curves in Fig. 1(a and b) show the unadjusted and adjusted concentration curves respectively for ANC visits in both 2008 and 2013 SLDHS.

The ANC concentration curve for 2013 lies slightly above the line of equality indicating that the poor made more ANC visits than the rich. On the other hand, the 2008 ANC concentration curve lies below and above the line of equality, suggesting that in 2008 there was little wealth related inequality in the number of ANC visits.

### Inequality analysis of PNC reviews

**Fig. 2:**
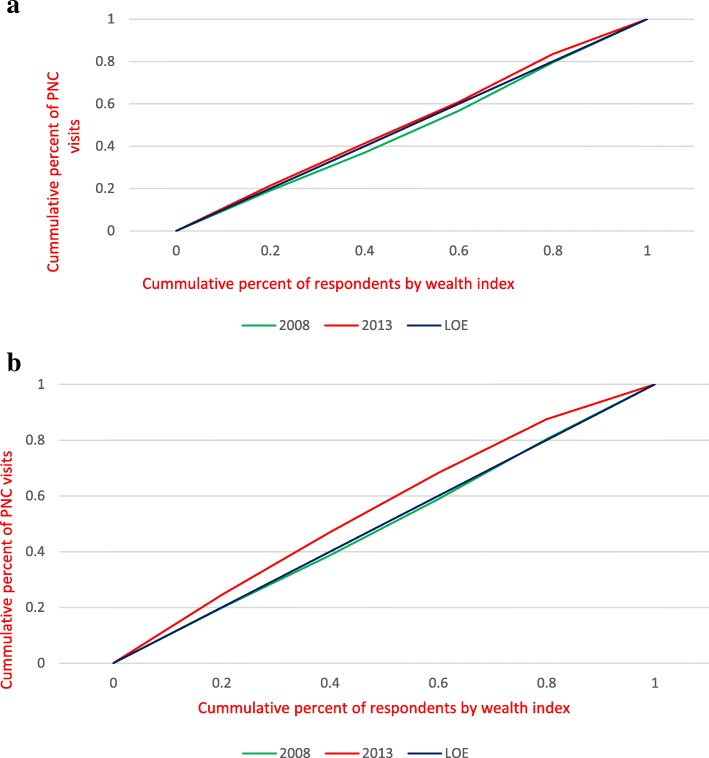
**a** Weighted Unadjusted Concentration Curves for PNC reviews in 2008 and 2013. **b** Weighted Adjusted Concentration Curves for PNC reviews in 2008 and 2013

Figure 2(a and b) show the unadjusted and adjusted concentration curves respectively for PNC reviews. In comparison with Figs. 1(a and b), unadjusted and adjusted concentration curves in Figs. 2(a and b) demonstrate that PNC reviews were more equally distributed in 2008 than ANC visits and this is evident in the values of concentration indices in 2008 for ANC visits and PNC reviews. Fig. 2(a and b) shows that in 2013, the poorest respondents ranked by wealth index utilized more PNC reviews than the richest.

Results of the inequality statistics for ANC visits and PNC reviews in 2008 & 2013 SLDHS are presented in Table 3. The differences in the adjusted concentration indices was statistically significant for ANC (t = 76.80, *p* <  0.001) and PNC (t = 4.84, *p* <  0.001) over the two survey periods.

**Table 3:** Test of Significance for Means & Standard Errors Obtained from Convenient Regressions

Antenatal Care Visits				
Unadjusted				
Year	2008	2013	Test statistic	*P* value
Estimate of concentration index	0.009612	0.002264	10.78	<0.001
Standard error	0.000584	0.000351		
Adjusted				
Year	2008	2013	Test statistic	*P* value
Estimate of concentration index	0.008331	-0.002263	76.80	<0.001
Standard error	0.000073	0.000030		
Postnatal reviews				
Unadjusted				
Year	2008	2013	Test statistic	*P* value
Estimate of concentration index	-0.000386	-0.001769	6.70	<0.001
Standard error	0.000133	0.000158		
Adjusted				
Year	2008	2013	Test statistic	*P* value
Estimate of concentration index	-0.001732	-0.001771	4.84	<0.001
Standard error	0.000007	0.000004		

### Determinants of institutional delivery

**Fig. 3:**
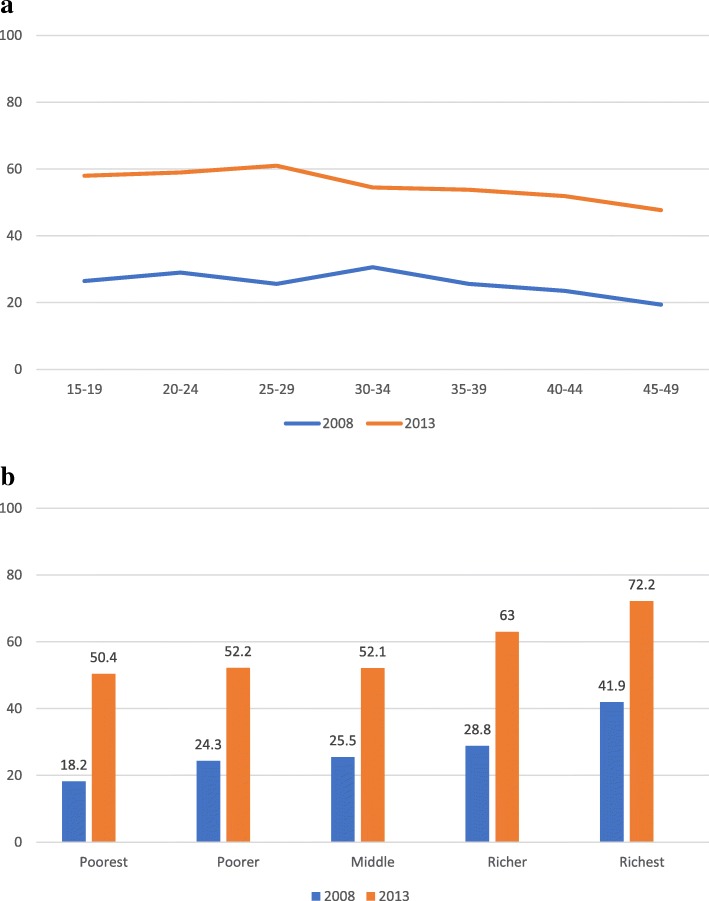
**a** Weight adjusted proportion of institutional delivery in 2008 and 2013 by Mother’s age group. **b** Weight adjusted proportion of institutional delivery in 2008 and 2013 by Economic status

In the 2013 SLDHS, institutional delivery coverage was 57.2% (Table 2), 50.4% among the poorest wealth quintile and 72.2% among the richest wealth quintile (Fig. 3b). Women in the richest wealth quintile were [AOR = 1.75; 95% CI (1.41, 2.17)] more likely to give birth at a health facility compared to women in the poorest wealth quintile (Table 4). The level of inequality in institutional delivery utilization increased, as the overall coverage increased, from a baseline utilization rate of 27% (Table 2). In 2008 SLDHS, the rate of institutional delivery was 18.2% among the poorest wealth quintile and 41.9% among the richest wealth quintile (Fig. 3b).Women in the richer wealth quantile were [AOR = 1.37;95%CI (1.05, 1.78)] times more likely to birth in a health facility compared to their poorest counterparts (Table 4a). .

**Table 4:** Adjusted and Unadjusted Odds Ratios of the Association between Demographic Characteristics of Women Respondents and Institutional Delivery in (a) 2008 SLDHS and (b) 2013 SLDHS

2008 SLDHS (a)					2013 SLDHS (b)			
Background Characteristic	Adjusted OR (95% CI)	*P*-value	Unadjusted OR (95% CI)	*P* value	Adjusted OR (95% CI)	*P*-value	Unadjusted OR (95% CI)	*P* value
Wealth index								
Poorest	1		1		1		1	
Poorer	1.49 (1.17, 1.89)	0.001	1.44 (1.14, 1.82)	0.002	1.22 (1.07, 1.40)	0.004	1.07 (0.94, 1.22)	0.290
Middle	1.52 (1.19, 1.93)	0.001	1.54 (1.22, 1.94)	< 0.001	1.28 (1.11, 1.47)	< 0.001	1.07 (0.94, 1.21)	0.335
Richer	1.37 (1.05, 1.78)	0.020	1.82 (1.45, 2.30)	< 0.001	1.72 (1.47, 2.00)	< 0.001	1.68 (1.47, 1.92)	< 0.001
Richest	1.30 (0.94, 1.80)	0.115	3.24 (2.57, 4.08)	< 0.001	1.75 (1.41, 2.17)	< 0.001	2.55 (2.20, 2.95)	< 0.001
Education Level								
None	1		1		1		1	
Primary	1.40 (1.13, 1.75)	0.002	1.78 (1.45, 2.18)	< 0.001	1.28 (1.12, 1.47)	< 0.001	1.37 (1.21, 1.56)	< 0.001
Secondary	1.98 (1.56, 2.52)	< 0.001	2.90 (2.38, 3.54)	< 0.001	1.76 (1.52, 2.03)	< 0.001	2.36 (2.09, 2.66)	< 0.001
Higher	3.76 (2.03, 6.96)	< 0.001	7.61 (4.27, 13.55)	< 0.001	3.40 (1.89, 6.11)	< 0.001	6.80 (3.88, 11.91)	< 0.001>
Occupation								
No	1		1		1		1	
Yes	1.20 (1.00, 1.43)	0.047	0.86 (0.73, 1.01)	0.066	0.72 (0.64, 0.81)	< 0.001	0.58 (0.52, 0.64)	< 0.001
Residence								
Urban	1		1		1		1	
Rural	0.47 (0.38, 0.58)	< 0.001	0.39 (0.34, 0.45)	< 0.001	0.68 (0.58, 0.80)	< 0.001	0.46 (0.42, 0.51)	< 0.001
Ethnicity								
Temne, Loko, & Limba	1		1		1		1	
Mende, Sherbro & Kono	2.22 (1.88, 2.63)	< 0.001	1.98 (1.69, 2.31)	< 0.001	3.08 (2.78, 3.41)	< 0.001	2.62 (2.39, 2.88)	< 0.001
Others	1.47 (1.19, 1.82)	< 0.001	1.53 (1.25, 1.87)	< 0.001	1.62 (1.41, 1.86)	< 0.001	1.58 (1.38, 1.80)	< 0.001
Religion								
Christianity	1		1		1		1	
Islam	0.87 (0.72, 1.04)	0.130	0.58 (0.49, 0.69)	< 0.001	0.86 (0.76, 0.97)	0.016	0.58 (0.52, 0.65)	< 0.001
Others	0.41 (0.15, 1.11)	0.079	0.17 (0.07, 0.46)	< 0.001	0.89 (0.37, 2.12)	0.791	0.56 (0.25, 1.26)	0.162
Mother’s age								
15–19	1		1		1		1	
20–24	1.19 (0.88, 1.61)	0.265	1.19 (0.89, 1.58)	0.249	1.09 (0.91, 1.30)	0.367	1.00 (0.84, 1.18)	0.976
25–29	1.04 (0.77, 1.40)	0.791	0.96 (0.72, 1.26)	0.749	1.25 (1.05, 1.50)	0.014	1.02 (0.87, 1.20)	0.841
30–34	1.25 (0.91, 1.71)	0.162	1.22 (0.91, 1.64)	0.179	1.03 (0.86, 1.25)	0.739	0.79 (0.67, 0.94)	0.006
35–39	1.08 (0.78, 1.49)	0.654	0.96 (0.71, 1.29)	0.775	1.06 (0.87, 1.29)	0.562	0.77 (0.64, 0.91)	0.003
40–44	0.99 (0.66, 1.49)	0.950	0.85 (0.58, 1.25)	0.404	1.05 (0.83, 1.33)	0.702	0.71 (0.57, 0.88)	0.002
45–49	0.96 (0.57, 1.64)	0.883	0.67 (0.40, 1.11)	0.116	0.80 (0.60, 1.06)	0.122	0.60 (0.46, 0.78)	< 0.001
Number of children	1.02 (0.99, 1.05)	0.119	1.02 (0.99, 1.05)	0.235	1.00 (0.99, 1.02)	0.823	1.00 (0.98, 1.02)	0.948

**Fig. 4:**
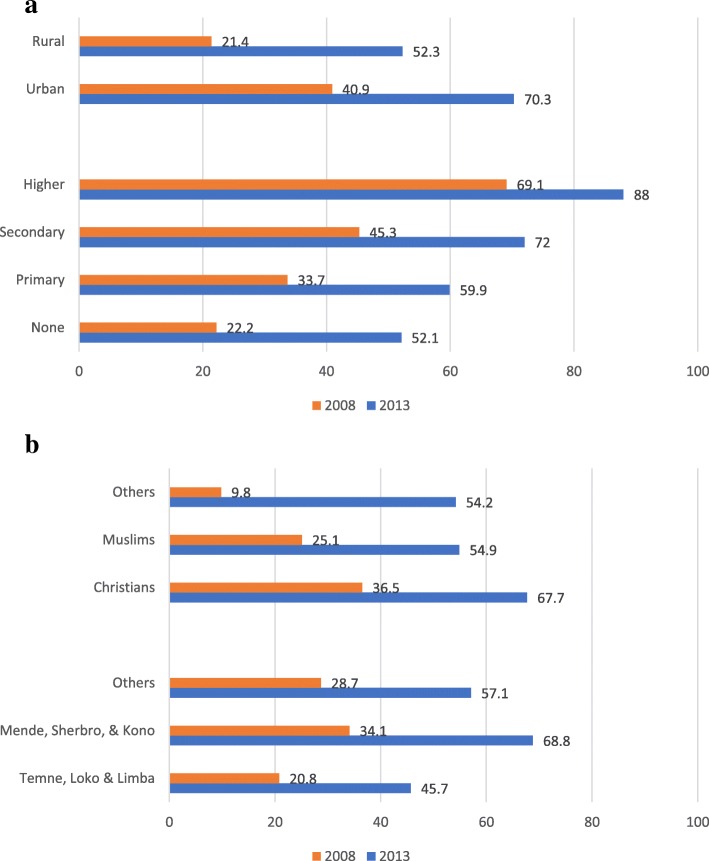
**a** Weight adjusted proportion of institutional delivery in 2008 and 2013 by Education level and Residence. **b** Weight adjusted proportion of institutional delivery in 2008 and 2013 by Ethnicity and Religion

The proportion of institutional delivery also varied significantly across education levels and residence. In 2008 SLDHS, 69.1% of women with higher than secondary school education had institutional delivery compared to the 22.2% of women with no education, representing a 46.9% difference in institutional delivery utilization rate (Fig. 4). Thus, women with higher than secondary school education were [AOR = 3.76; 95% CI (2.04, 6.94)] times more likely to give birth at a health facility compared to women with no formal education (Table 4a). In 2013 SLDHS, the overall coverage for institutional delivery improved for all education levels and the inequality gap narrowed. Women with higher than secondary school education had 88% institutional delivery rate compared to the 52.1% of those with no formal education, representing a 35.9% difference in institutional delivery utilization rate (Fig. 4). Women with higher than secondary school education in 2013 were [AOR = 3.4; 95% CI (1.89, 6.11)] times more likely to birth at a health facility compared to women with no formal education (Table 4b).

Institutional delivery was lowest among women in rural settings (21.4% & 52.3% in 2008 & 2013 respectively) compared to their counterparts in urban settings (40.9% & 70.3% in 2008 and 2013 respectively) (Fig. 4). Women in rural areas were 32% less likely in 2008 [AOR = 0.68; 95% CI (0.58, 0.80)] and 53% less likely in 2013 [AOR = 0.47 [95% CI (0.37, 0.58)] to give birth at a health facility compared to their counterparts in urban settings (Table 4).

The institutional delivery rate varied significantly across ethnic and religious subgroups of the respondents in both 2008 and 2013 SLDHS. In 2013 SLDHS, women from tribes found predominantly in the South and South-east of the country (Mende, Sherbro & Kono) had 23.1% more utilization rate of institutional delivery than women from tribes predominantly located in the Northern and Western parts of the country (Temne, Loko & Limba). A 13.3% difference in institutional delivery rate in 2008 between tribes in the South and Southeast and those in Northern and Western of the country (Fig. 4b). Women from tribes in the South & South-eastern regions were more likely to birth at a health facility compared to their counterparts from tribes in the Northern & Western parts of the country in 2013 [AOR = 3.08;95% CI (2.78, 3.42)] and 2008 [AOR = 2.22; 95% CI (1.88, 2.63)] (Table 4).

The percentage point difference between Christian and Muslim women in the utilization rate of institutional delivery was 11.4% in 2008, and this difference increased to 12.8% in 2013 (Fig. 4b). Muslim women in 2013 were 14% less likely [AOR = 0.86; 95% CI (0.76, 0.97)] to deliver at a health facility compared to their Christian counterparts (Table 4a).

We observed a significant difference in the unadjusted (t = 1.80, *p* = 0.036) and adjusted (t = 1.73, *p* = 0.042) odds ratios of the richest-poorest subgroups of society with regards to the utilization of institutional delivery in 2008 & 2013 (Table 5). This represents a significant gap in wealth related inequality in institutional delivery utilization between the rich and the poor over the study period.

**Table 5:** Test of Equality of the Odds Ratios obtained from Binomial Logistic Regression

Institutional Delivery^a^				
Unadjusted				
Year	2008	2013	Test statistic	*P* value
Rich-poor odds ratio	3.24	2.55	1.80	0.03593
Standard error of the odds ratio	0.340	0.177		
Adjusted				
Year	2008	2013	Test statistic	*P* value
Rich-poor odds ratio	1.3	1.75	1.73	0.04182
Standard error of the odds ratio	0.186	0.173		

^a^All values were obtained at a 95% confidence interval

## Discussion

.Our results show changes in distribution of utilization of MCH services across wealth quintiles over time alongside a significant increase in the proportion of women eligible for free MCH services utilizing such services before and 3 years after the introduction of FHCI in Sierra Leone. We found that while utilization of ANC was unequally distributed to the advantage of the richest women prior to FHCI, it was unequally distributed to the advantage of the poorest women in 3 years after the introduction of the FHCI in 2013

. This finding is consistent with a similar study in Afghanistan [30] but inconsistent with many published studies elsewhere [31–35]. The observed inconsistency may have arisen from minor differences in variable definition [31–35] variable types [32, 34] included the use of cross sectional study data with much shorter periods and not DHS by others [31, 34]. It may also be due to differences in the economic profile and health systems of the different countries [32–35] or the use of single DHS dataset as opposed to a time trend review [35].

Our findings have also demonstrated that PNC reviews which were slightly unequally distributed in favor of the poor in 2008 were in 2013 significantly unequally distributed in favor of the poor suggesting that other important factors besides wealth may be at play. Children of poorer households are more likely to get sick than those of richer households [36, 37] and that poorer women are more likely to be fertile [38, 39], therefore increasing health needs among this group. Trends in health seeking behavior in the country may play a role. People tend to utilize the informal healthcare before seeking the formal sector as a form of last resort [40–42] and recent Sierra Leonean studies suggest that pregnant women, lactating mothers and infertile women practice medical pluralism [43–45].. Therefore that may be the reason why mild to moderates ailments may not warrant PNC reviews utilization within richer households. Our finding is inconsistent with findings in Ghana and other published literature globally [35, 46, 47]. The inconsistency of our finding with the globally literature may reflect the unique cultural, political and social context of Sierra Leone. The inconsistency may be due to differences in the data source used and the analytical approaches. For instance, while Ghana shares a similar cultural profile to that of Sierra Leone, the study examining the impact of the free user policy on utilization analyzed data from the Ghana Maternal Health Survey 2007 [48]. The difference in findings may also be reflective of differences in health policy to healthcare delivery design. For example, in Bangladesh [35] user fees were abolished alongside the implementation of a sector wide approach (SWAp), which resulted in.the narrowing the gap in wealth-related inequity between the rich and the poor.

We found that wealth-related inequality in the utilization of health facilities for delivery increased over the study period to the disadvantage of the poor. Our finding is consistent with other studies, which reported increasing wealth related inequity in institutional delivery between the poor and the rich. [30–32, 46, 49, 50]. The observed increase in wealth related inequality in utilization of institutional delivery that favors the rich may be a significant pointer to the fact that poor and less educated women view the conventional healthcare setting as a hostile environment that is not culturally sensitive to the needs of women delivering at these institutions. For example, women in rural areas prefer to squat during delivery unlike the lithotomy position promoted in health facilities [51]. Some tribes have specific rituals observed around the time of delivery, such as burial of the placenta by specific family members or its consumption as food, which may not be accommodated in the healthcare setting [52, 53]. Also, women of secret traditional societies do not prefer to be attended to during delivery by women who are not members of these secret societies or worse still by men [54]. The healthcare delivery system may therefore need to re-think its approach and re-evaluate its policies to providing institutional delivery to accommodate the legitimate concerns of women and therefore promote institutional delivery, which is key to reducing the current high maternal and infant mortality in Sierra Leone.

We found that despite an encouraging decrease in home delivery rates from 73% in 2008, the 43% delivery rate in 2013 remains high, which may indicate barriers beyond a policy of free services. Our results showed higher levels of education and urban residence have a relationship to utilization of MCH services, consistent with other evidence [31, 35, 55, 56]. The influence of residence on inequality in the utilization of MCH services may be due to the availability of more health facilities in urban settings than in rural settings [57] . In addition, rural health facilities are usually under staffed and this may serve as a disincentive to seeking MCH services in rural residences [58]. Tackling such inequitable distribution of health facilities and addressing the human resource for health gap is a fundamental goal in the free health care initiative [13]. Residents in rural settings hold strong cultural beliefs that limit their seeking of institutional delivery such as that labor is a normal process that can only requires hospitalization and surgery for weak women or those who have invited a curse upon themselves [59].

### Policy and practice implications

FHCI has been successful at increasing utilization of MCH services over time, but the serious gaps in equity of utilization of services across different wealth quintiles remain problematic. In addition, the increase in inequity of utilization of facility-based delivery services, a factor with strong correlation to maternal mortality [49], among the poorest women, warrants immediate action to ensure that policies are benefitting all levels of society in order to achieve universal health coverage. In order for Sierra Leone to meet its commitment to achieving SDG3, a review of the implementation strategies supporting the FHCI with specific reference to equity is required. Such a review should include consideration of implementation approaches to address specific equity gaps. Bangladesh has shown that a sector-wide approach (SWAp) that harnesses the significant inputs of other sectors such as agriculture, infrastructure, education, and traditional leadership, has promise [35]. Such an adaption of “Health in all Policy” approach allows for developments in the agricultural sector to enhance the rural incomes, thus helping to address indirect costs of accessing free services [60]. Interventions that address quality of care in relation to delivery services, with a specific focus on accommodating social and cultural preferences of the poorest women, should be considered. Similarly, investments in, strategic deployment of, and retention of human resources for health in rural and remote communities is needed to create a more balanced and fair of services. Finally, strategies to understand and target services preferences, health promotion needs, and other barriers to accessing institutional delivery services for the poorest, uneducated, and/or rural women and their families should be reviewed.

### Limitations

Our study has several limitations. Our methods limit our ability to attribute causality in the changes in MCH utilization and distribution across wealth quintiles over time to FHCI. The lack of a comparison group means that this study cannot rule out the contribution of other factors to the recorded incremental changes in the utilization of MCH services. Recall bias on events that happened within the 5 years prior to each survey is another limitation. The inclusion of only women who gave birth to their last child in the 5 years prior to each survey may have reduced the number of eligible women from the richest wealth quintile who are known to be less willing to give birth to more kids. This effect is however expected to be minimal and is counteracted by the exclusion of women whose children died within the first 2 months for PNC reviews since infant mortality is more common among the poor. However, this study may be one of the first in Sierra Leone to utilize the DHS in the evaluation of the impact of FHCI on inequity of MCH services across wealth quintiles.

## Conclusion

Although it is difficult to draw a conclusive causal link between the increase in the utilization rate of the selected MCH services and the free healthcare initiative, it appears that the initiative is at the least not pro-rich for ANC visits and PNC reviews. Steps need to be taken to address the growing wealth related inequality to the disadvantage of the poor that accompanies the overall increase in institutional delivery rate. Pronounced level of inequality in institutional delivery was also linked with women level of education and residence, revealing that women with no formal education or residents in rural settings were the most underserved subpopulations. It is obvious that in addition to wealth differences, other sociodemographic characteristics like education level, residence, ethnicity, and religion contribute to the existing inequities. Promoting the education level of women and increasing the number of qualified staff at health facilities in rural settings, and ensuring culturally sensitive, quality care should be prioritized to improve the odds against socioeconomically disadvantaged women.

## Additional files

Additional file 1: Number of women interviewed and included in the final analysis in the 2008 and 2013 SLDHS. An algorithm of the number of women interviewed and included in the final analysis for antenatal care (ANC), postnatal care (PNC) and place of delivery (PLOD) in the 2008 and 2013 SLDHS. (DOCX 42 kb)

Additional file 2: Dependent variables. Operational definitions of the dependent variables. (DOCX 14 kb)

Additional file 3: Independent variables. Operational definitions of the independent variables. (DOCX 15 kb)

## Data Availability

The dataset for this study can be access from the DHS program-ICF International, Rockville, data after the submission of a written request. It is available at https://dhsprogram.com/data/available-datasets.cfm

## Abbreviations

ANC: Antenatal care
FHCI: Free healthcare initiative
MCH: Maternal child health
MDG: Millennium development goals
PLOD: Place of delivery
PNC: Postnatal care
SDG: Sustainable development goals
SLDHS: Sierra Leone Demography Health survey
SWAp: Sector wide approach
