# Supplementary material for: Impact of the free healthcare initiative on wealth-related inequity in the utilization of maternal & child health services in Sierra Leone
Source: BMC Health Serv Res. 2019 Jun 3;19:352. doi: 10.1186/s12913-019-4181-3 (PMC6547484; doi:10.1186/s12913-019-4181-3)
Supplement: Supplementary file 1 — Number of women interviewed and included in the final analysis in the 2008 and 2013 SLDHS. An algorithm of the number of women interviewed and included in the final analysis for antenatal care (ANC), postnatal care (PNC) and place of delivery (PLOD) in the 2008 and 2013 SLDHS. (DOCX 42 kb) [file 12913_2019_4181_MOESM1_ESM.docx]

**Additional file 1**

7374 women interviewed in 2008

**7374** women interviewed in 2008

**16658** women interviewed in 2013

**8011** cases excluded either because they had no child or no last child delivered within the five years prior to the survey

**3271** cases excluded either because they had no child or no last child delivered within the five years prior to the survey

**4103** included in the analysis of ANC & ID

**8647** included in the analysis of ANC & ID

**277** cases excluded because their last child died within the first two months of life

**152** cases excluded because their last child died within the first two months of life

**8370** included in the analysis of PNC reviews

**3951** included in the analysis of PNC reviews

Exclusion of women who responded don’t know or who had missing information

Exclusion of women who responded don’t know or who had missing information

**7478, 8625** & **7971** women included in the final analysis of ANC, ID & PNC respectively

**3346, 4053** & **3504** women included in the final analysis of ANC, ID & PNC respectively
