# Supplementary material for: Impact of the free healthcare initiative on wealth-related inequity in the utilization of maternal & child health services in Sierra Leone
Source: BMC Health Serv Res. 2019 Jun 3;19:352. doi: 10.1186/s12913-019-4181-3 (PMC6547484; doi:10.1186/s12913-019-4181-3)
Supplement: Supplementary file 2 — Dependent variables. Operational definitions of the dependent variables. (DOCX 14 kb) [file 12913_2019_4181_MOESM2_ESM.docx]

**Additional file 2**

**Dependent variables**

| **Scale** | **Source** | **Information** | **Dependent Variable** |
| --- | --- | --- | --- |
| Numerical and discrete  Converted into three subcategories (none, up to four and more than four visits) | Response of interviewees | Consulting a skilled health care provider during pregnancy for pregnancy related issues | Antenatal Care Visits |
| Categorical, nominal and dichtomous into   - no - yes | Response of interviewees | Location of birth of baby | Institutional delivery |
| Numerical and discrete  Converted to categorical nominal variable [complete(≥4) vs incomplete(<4)] | Response of interviewees | Clinical review of mother and baby post-delivery by a skilled health care provider. A composite variable | Post-natal care reviews |
