# Supplementary material for: Impact of the free healthcare initiative on wealth-related inequity in the utilization of maternal & child health services in Sierra Leone
Source: BMC Health Serv Res. 2019 Jun 3;19:352. doi: 10.1186/s12913-019-4181-3 (PMC6547484; doi:10.1186/s12913-019-4181-3)
Supplement: Supplementary file 3 — Independent variables. Operational definitions of the independent variables. (DOCX 15 kb) [file 12913_2019_4181_MOESM3_ESM.docx]

**Additional file 3**

**Independent variable**

| **Scale** | **Source** | **Information** | **Independent Variable** |
| --- | --- | --- | --- |
| Categorical and ordinal | Response of participants and observations made by investigators during the interview | Wealth is determined by scoring households based on a set of characteristics, including access to electricity and ownership of various consumer goods | Wealth index |
| Categorical and ordinal into   - No education - Primary (0-6) - Secondary (7-12) - Higher (>12^th^ level) | Response of interviewee | Refers to the highest level of education attended by interviewee | Education level |
| Categorical and nominal into   - Yes - No | Response of interviewee | Refers to the possession of a paid job by interviewee | Occupation |
| Categorical and nominal into   - Urban - Rural | Response of interviewee | De facto place of residence | Residence |
| Categorical and nominal into   - Temne, Loko & Limba - Mende, Shebro & Kono - Other Sierra Leonean and Non Sierrra Leonean interviewee | Response of interviewee | Identifying with one of the tribes of Sierra Leone | Ethnicity |
| Categorical and nominal into   - Christianity - Islam - Others | Response of interviewee | Professing belief in the tenets of a specific faith | Religion |
| Categorical and ordinal | Response of interviewee | Length of time lived in years | Mother’s age |
| Numerical and discrete | Response of interviewee | Number of elder children born by the same mother | Number of siblings |
